# Forkhead Box Q1 Is Critical to Angiogenesis and Macrophage Recruitment of Colorectal Cancer

**DOI:** 10.3389/fonc.2020.564298

**Published:** 2020-11-30

**Authors:** Hui Tang, Ji Zheng, Xuan Bai, Ke-Lin Yue, Jian-Hua Liang, Dan-Yang Li, Lin-Ping Wang, Jin-Li Wang, Qiang Guo

**Affiliations:** ^1^ Yunnan Digestive Endoscopy Clinical Medical Center, Department of Gastroenterology, The First People’s Hospital of Yunnan Province, Kunming, China; ^2^ Medical Faculty, Kunming University of Science and Technology, Kunming, China; ^3^ Genetic Testing Center, Qingdao Women and Children’s Hospital, Qingdao, China

**Keywords:** FOXQ1, colorectal cancer, tumor angiogenesis, macrophages, tumor microenvironment

## Abstract

Angiogenesis and the tumor microenvironment (TME) play important roles in tumorigenesis. Forkhead box Q1 (FOXQ1) is a well-established oncogene in multiple tumors, including colorectal cancer (CRC); however, whether FOXQ1 contributes to angiogenesis and TME modification in CRC remains largely uncharacterized. Here, we demonstrate an essential role of FOXQ1-induced angiogenesis and macrophage recruitment in CRC that is related to its ability to promote the migration of endothelial cells and macrophages through activation of the EGF/PDGF pathway and the Twist1/CCL2 axis. We also provide evidence showing that the clinical significance between FOXQ1, Twist1, CCL2, and macrophage infiltration is associated with reduced 8-year survival in CRC patients. Our findings suggest FOXQ1 plays critical roles in the malignancy and progression of CRC, Therefore, FOXQ1 may serve as a therapeutic target for inhibiting angiogenesis and reducing macrophage recruitment in CRC.

## Introduction

Tumor initiation and malignancy are closely associated with angiogenesis (1), and pathological neovascularization initiates tumor tissue ischaemia, growth, and metastasis (2). In angiogenesis, endothelial cell (EC) proliferation and migration cause new capillaries to develop from preexisting capillaries (3). In addition, macrophages within the tumor microenvironment (TME) facilitate angiogenesis and extracellular-matrix breakdown, thus remodelling and promoting tumor cell migration, invasion, and metastasis (4). Angiogenesis involves complex signalling pathways and is associated with the production of many key regulatory factors, such as epidermal growth factor (EGF)/platelet-derived growth factor (PDGF) (5), vascular endothelial growth factor (VEGF), epidermal growth factor-like growth factor (HB-EGF) (6), angiopoietins, tissue inhibitor of metalloproteinases 1 (TIMP-1), and basic fibroblast growth factor (bFGF) (7). Therefore, angiogenesis and the inflammatory microenvironment play important roles in tumorigenesis.

Forkhead box Q1 (FOXQ1) is a member of the forkhead transcription factor family (8) with demonstrated functional roles in hair follicle morphogenesis and gastric epithelial differentiation (9). Studies have indicated that FOXQ1 is also an oncogene in multiple tumors, including colorectal cancer (CRC) (10–12), non-small cell lung cancer (13), breast cancer (14, 15), ovarian cancer (16), bladder carcinoma (17), stomach cancer (18), liver cancer (19), and neuroglioma (20). Recent studies suggest that the tumorogenic function of FOXQ1 may be related to its ability to promote cell cycle progression (10, 16), tumor angiogenesis (10), cell proliferation (11, 21), stem cell-like properties (14), resistance to chemotherapy-induced apoptosis (14), modification of the TME (19), epithelial-mesenchymal transition (EMT) (22, 23), senescence-associated inflammation (24), and Wnt signaling activation (25). We previously demonstrated that aberrant expression of FOXQ1 is correlated with metastasis in CRC (12). Furthermore, FOXQ1 has been shown to be a regulator of cancer invasion and metastasis in CRC and a modulator of Twist1 expression (11). The role of Twist1-induced CCL2 in angiogenesis has been demonstrated (26), which raises the possibility that FOXQ1 may induce angiogenesis in CRC by inducing Twist1; however, a role for FOXQ1 in inducing tumor angiogenesis and TME modification in CRC has not been evaluated.

In this study, we demonstrate that FOXQ1 promotes angiogenesis in CRC cells by activating the expression of angiogenic factors while reducing the expression of angiogenic inhibitors. In addition, FOXQ1 can promote recruitment of macrophages by activating the Twist1/CCL2 axis. Our results indicate that FOXQ1 overexpression correlates clinically with overall survival in CRC, suggesting that it might serve as a new target for anti-angiogenic and anti-inflammation therapy.

## Materials and Methods

### Cell Culture

The human CRC cell lines LS174T, Colo320, SW480, HCT116, DLD1, HT29, and LoVo; HEK293 cells; and human macrophage U937 cells were purchased and authenticated from the Cell Bank of the Chinese Academy of Science in Shanghai, China. HUVECs were purchased from Yingrun Biotechnology Co. Ltd. in Changsha, China. All cells were cultured as recommended by the manufacturer.

### Plasmid Construction and Transfection

Three siRNAs targeting the human FOXQ1 sequence (NM_033260.3) were designed using the siRNA Target Finder (InvivoGen, San Diego, CA, USA), and one scrambled siRNA was designed as a negative control (**Table S1**). The corresponding primers used for plasmid construction were synthesized by Sangon (Shanghai, China) (**Table S2**) and then ligated to the lentiviral PLKO.1 vector. A FOXQ1 cDNA plasmid purchased from GeneChem (Shanghai, China) was cloned into a pLVX-IRES-Puro lentiviral vector, and the recombinant plasmid was named lv-FOXQ1. Lentiviral vectors and packing vectors (pRSV-rev, pMDlg-pRRE and pCMV-VSV-G) were co-transfected into HEK293 cells. Lentivirus was collected to infect DLD1 and HCT116 cells. Stable cells were generated after selection with puromycin (12 mg/ml) (Solarbio, Beijing, China) for 20 days. The most effective knockdown/overexpression cells were designated DLD1-shFOXQ1/HCT116-FOXQ1, and the corresponding controls were named DLD1-shControl/HCT116-vec. For transient transfection, either siRNA targeting Twist1 or vector overexpressing Twist1 (pcDNA3.1-Twist1), or the respective controls (si-Scramble and pcDNA3.1), were purchased from GeneChem (Shanghai, China) and transfected into cells using Lipofectamine 2000 according to the manufacturer’s instructions.

### RNA Isolation and Quantitative Real-Time PCR (qRT-PCR) Analysis

RNA was extracted using TRIzol reagent (Invitrogen, Carlsbad, CA, USA) and was reverse transcribed to cDNA using the GoScript Reverse Transcription System (Promega, Madison, WI, USA). qRT-PCR was performed using the LightCycler 480 (Roche, USA) with SYBR Premix Ex Taq II (Takara, China). Each sample was analysed in duplicate with GAPDH as a reference. Oligonucleotide sequences are provided in supplemental file 1 (**Table S3**). Quantitative results were calculated using the 2^-△△CT^ method.

### Transcriptional Signature Analysis

Transcriptional signatures of DLD1-shFOXQ1 and DLD1-shControl cells were obtained and compared using the Human GE 4 × 44K Microarray platform (Agilent Technologies, Santa Clara, CA, USA). Microarray experiments were performed by KangChen Bio-tech, Shanghai, China. Quantile normalization and subsequent data processing were performed using the GeneSpring GX v11.5.1 software package (Agilent Technologies, Santa Clara, CA, USA). Differentially expressed genes (DEGs) with statistical significance were identified by volcano plot filtering. KEGG pathway analysis was performed using the NIH gene annotation software DAVID.

### Western Blot Analysis

Cells were washed with PBS and lysed in ice-cold lysis buffer containing protease inhibitor cocktail (Sigma-Aldrich, St. Louis, MO, USA) for 30 min. Lysates were separated by electrophoresis and transferred onto polyvinylidene difluoride membranes (Millipore; Bedford, MA, USA). The membranes were blocked and incubated with primary antibodies. Antibodies against FOXQ1 (ab51340), PDGF (ab178409), HB-EGF (ab92620), and Twist1 (ab175430) were purchased from Abcam (England). The antibody against EGFR (3265S) was purchased from Cell Signaling (Cold Spring Harbor, NY, USA), and antibodies against ANG (18302-1-AP), PDGFRB (13449-1-AP), ANGPT1 (23302-1-AP), PLAUR (10286-1-AP), tPA (10147-1-AP), VEGF (19003-1-AP), and β-actin (66009-1-Ig) were purchased from Proteintech (Wuhan, China). β-Actin was used as the loading control. The immunoreactive proteins were visualized with SuperSignal West Dura Chemiluminescent Substrate (ThermoFisher, Waltham, MA, USA).

### Cell Proliferation Assay and Clone Formation Assay

The DLD1-shFOXQ1 and DLD1-shControl cells (2,000 cells/well in a 96-well plate) were incubated with medium containing 10% FBS at 37°C for 24 h, 48 h, 72 h, and 98 h, respectively. At the end of incubation, 10 μl Cell Counting Kit-8 (CCK-8) solutions (Beyotime Biotech, Shanghai, China) were added and incubation for another 4 h at 37°C, and then OD_450nm_ was measured by Microplate Reader (BioTek, Winooski, VT, USA). For clone formation assay, 500 cells were seeded into 6-well plates and incubated for 14 days (with medium replaced every three days). Then, the cells were fixed and stained with 1% crystal violet at room temperature for 20 min. Photos were taken and the number of clones was counted.

### Cell Migration Assay and Wound Healing Assay

Analysis of cell migration was done by using Transwell insert with 8.0 μm membrane pores (BD, San Jose, CA, USA) according to the manufacturer’s protocol. Migration was additionally evaluated with the wound healing assay. Briefly the DLD1-shFOXQ1 and DLD1-shControl cells were seeded in 24 well plates at a density that enabled a confluency of 80% to be attained 24 h after plating. A 200 μl filter tip was used to gently scratch the cell monolayer across the center of the well. The cells were then gently washed with PBS to remove the dislodged cells, and then replenished with fresh medium, after which the first images were acquired. The cells were incubated for a further 24 h after which a second set of images were acquired to determine the extent of wound closure.

### Preparation of Conditioned Medium (CM) From CRC

HCT116-FOXQ1 and DLD1-shFOXQ1 cells were cultured in RPMI-1640/5% FBS medium. At 90% confluence, the culture medium was switched to either serum-free GT-T551 (Takara, Dalian, China) or fresh RPMI-1640/5% FBS medium and was incubated for 48 h before the CM was collected. Serum-free GT-T551 CM was collected for protein array analysis, and RPMI-1640/5% FBS CM was collected for EC migration assay and ELISA.

### EC Migration Assay

The HUVEC migration assay was performed as described previously with minor modifications (27). HUVECs are regarded as a cell model in angiogenesis assays *in vitro*, for which they have been widely used to detect tube formation abilities on matrigel (28, 29). HUVECs at passage 5 or less were serum-starved for 5 h in serum-free EBM-2 (Lonza Cologne, Walkersville, MD, USA) supplemented with 2 mM L-glutamine, 100 U/ml penicillin, 100 ng/ml streptomycin, 10 ng/ml heparin, and 0.1% FBS. On the following day, a Transwell migration assay was performed using BD cell culture inserts (3.0 μm membrane pores) according to the manufacturer’s protocol. Transwells were assembled in 12-well plates, and the lower chambers were filled with 1,500 μl of medium containing 50% fresh EBM-2/10% FBS and 50% CRC-CM. Then, 60,000 HUVECs resuspended in 500 μl serum-free EBM-2 were inoculated onto the upper chamber of each Transwell, and the plates were placed at 37°C in a 5% CO_2_ incubator for 4 or 8 h. After removing the non-migrating cells with a cotton swab, the cells that had migrated to the lower surface of the filters were fixed with cold 4% paraformaldehyde and stained with 0.1% crystal violet/20% (v/v) methanol. Then, the migrated cells on the bottom of the Transwell inserts were counted. All assays were performed in triplicate. Three random fields were chosen for each insert, and the cells were counted and imaged under a light microscope.

### ELISA

To measure CCL2 secretion, CM from CRC cells cultured for 48 h was collected, and CCL2 concentrations were measured using the CCL2 ELISA detection kit (eBioscience, Houston, Texas, USA) according to the manufacturer’s protocol.

### Microvessel Morphogenesis Assay

A microvessel formation assay was performed as described previously with minor modifications (27). HCT116-FOXQ1, DLD1-FOXQ1-shRNA cells, and HUVECs were cultured in reduced serum conditions in RPMI 1640 medium containing 1% FBS for 12 h, and then 2 x 10^5^ HUVECs were mixed with either 2 x 10^5^ HCT116-FOXQ1 or 2 x 10^5^ DLD1-shFOXQ1 cells and grown in 24-well plates precoated with growth factor-reduced Matrigel basement membrane matrix (BD Biosciences, San Jose, CA, USA). After 20 h incubation, images were taken, and microvessel formation abilities were quantified by measuring the cumulative tube length using ImageJ software (NIH, Bethesda, MD, USA). The number of intact or damaged microvessels was quantified. For the CM experiment, HCT116-FOXQ1 and DLD1-FOXQ1-shRNA cells were cultured in complete medium containing l0% FBS for 48 h, and then cell culture supernatants were collected as CM. At the same time, HUVECs were cultured in reduced serum conditions in RPMI 1640 medium containing 1% FBS for 12 h. Then, 2 × 10^5^ HUVECs were resuspended in either the HCT116-FOXQ1 CM or DLD1-shFOXQ1 CM and grown in 24-well plates precoated with Matrigel. Microvessel formation was documented after 20 h by microscopy (Axiovert 200; Carl Zeiss, Gttingen, Germany).

### Tumor Xenograft Model and *In Vivo* Angiogenesis Assay

Female BALB/c nude mice (6–7 weeks old) were purchased from SLAC Laboratory Animal Co., Ltd. (Shanghai, China). Ten mice were randomly divided into two groups. A total of 200 µl of a 3:1 mixture of growth factor-reduced Matrigel (BD Biosciences, San Jose, CA, USA) and 1 × 10^7^ DLD1-shFOXQ1 or DLD1-shControl cells in DMEM were injected into the dorsal flank of each mouse. The date at which the first grossly visible tumor appeared was recorded, and the tumor size was measured every 3 days thereafter. Mice were sacrificed on day 16, and tumors were dissected and fixed for histological examination and microvessel density (MVD) analysis. The animal study was reviewed and approved by Institutional Animal Care and Use Committee, the First People’s Hospital of Yunnan Province (Yunnan, China).

### Immunohistochemistry and MVD Analysis

Antibodies against FOXQ1 (ab51340), CD31 (ab28364), CD34 (ab81289), and F4/80 (ab6640) were purchased from Abcam (England), and paraffin-embedded mouse tumor serial sections (4 μm) were stained with anti-FOXQ1 antibody to confirm the efficiency of FOXQ1 gene knockdown. Haematoxylin and eosin (H&E) staining was performed to verify the morphological characteristics of xenograft tumor tissues. Anti-CD31 and anti-CD34 antibodies were used for EC staining. The macrophage content was measured by staining for the mature macrophage marker F4/80. For MVD analysis, CD31^+^ or CD34^+^ blood vessels in tumor sections were counted in 10 random fields (hpfs, 400×) in vascular hot spots, as previously described (30). For macrophage quantification, five random fields in F4/80^+^ hot spots were scored on a scale of 0-6 for staining intensity and distribution within a field: 0, undetectable; 1, faint, discrete patches; 2 faint, all over; 3 medium, discrete patches; 4 medium, all over; 5 intense, discrete patches; 6 intense, all over. The images were documented by two pathologists blinded and processed using Photoshop CS4 (Adobe Systems Incorporated, San Jose, CA, USA).

### Protein Array Analysis

HUVECs at passage 5 or less were seeded at a density of 10,000 cells per well in a 12-well plate and then switched for 48 h to RPMI-1640/5% FBS medium. Then, the RPMI-1640/5% FBS medium was replaced with medium consisting of 50% RPMI-1640/5% FBS and 50% CRC-CM and cultured for another 48 h. The cells were lysed in NP-40 lysis buffer (Beyotime, Beijing, China) containing a protease inhibitor cocktail (Promega, Madison, WI, USA). CM of CRC cells and cell lysates of HUVECs were then assayed for angiogenesis factor levels by using protein arrays (QAH-ANG-2 and QAH-ANG-3, Quantibody Human Angiogenesis Array, RayBiotech, Norcross, GA, USA), which can quantitatively measure 60 well-established angiogenic proteins by comparing fluorescent signals to the standard curve. Analysis was performed according to the manufacturer’s instructions, and the detected signals were quantified using a gel documentation system (UVItec, Cambridge, MA, USA).

### *In Vitro* Macrophage Cell Migration Assay

U937, a human monocytic cell line, was differentiated into macrophages by using 100 nM phorbol 12-myristate 13-acetate (PMA; Sigma-Aldrich, St. Louis, MO, USA, P8139) as described previously (31). Then, macrophage migration assays were performed as described (32). Briefly, macrophages were seeded (5 × 10^4^ cells/insert) onto the upper well of Transwell inserts with 8.0 μm membrane pores (BD, San Jose, CA, USA) for 2 h to allow attachment to the membrane, and then Transwells were moved to 24-well plates containing 0.7 ml CM either from HCT116-FOXQ1 (co-transfected either with si-Twist1 or si-Scramble) or from DLD1-shFOXQ1 (co-transfected either with pcDNA3.1-Twist1 or pcDNA3.1) and further incubated for 4 h. Cells in the upper chamber were removed with a cotton swab after fixation in 4% paraformaldehyde and stained with 0.1% crystal violet, while the migrated macrophages in the lower chamber were quantified using 12–15 random fields. Three independent experiments were performed.

### CRC Tissue Microarray

Tissue microarrays containing a total of 90 pairs of colorectal tumor tissues and matched adjacent normal tissues, together with pathological staging data in accordance with TNM classification of the American Joint Committee on Cancer (2010) and follow-up survival time after surgery, were obtained from Shanghai Biochip Co., Ltd., Shanghai, China (HCol-Ade180Sur-06). Antibodies against FOXQ1 (ab51340), CD31 (ab28364), Twist 1 (ab175430), CCL2 (ab73680), and CD68 (ab955) were purchased from Abcam (Cambridge, MA, USA), and tissue microarray analysis was performed using a standard immunohistochemistry protocol. The median value of the immunoreactivity score (IRS) was chosen as the cut-off for high and low protein expression levels based on a measure of heterogeneity according to the log-rank test with respect to disease-specific survival (DSS), as described previously (33). Cut-off values for the scoring system were assigned as follows: high expression of FOXQ1, Twist1, and CCL2 were defined as an IRS of ≥ 4 (4, 6, 8, 9, and 12), and low expression was defined as an IRS of < 4 (0, 1, 2, and 3). High expression of CD31 and CD68 was defined as an IRS of ≥ 150 and of ≥ 400, respectively, and low expression was defined as an IRS of <150 and of <400, respectively. Immunostained sections were scanned using a microscope (Axiovert 200; Carl Zeiss, Gttingen, Germany). Data for seven patients were excluded because the dots were off the chips during the experiment. Data for a total of 83 patients with CRC were therefore included in the final analysis.

### Statistical Analysis

Statistical analyses were performed with GraphPad Prism 6.01 and SPSS v.19. An unpaired two-tailed Student’s *t*-test was performed for two-group comparisons, and one-way analysis of variance (ANOVA) was performed for multiple group comparisons. Survival curves were calculated using the Kaplan-Meier algorithm and log-rank test.

## Results

### Transcriptional Signature Analysis of CRC Cells With FOXQ1 Overexpression or Knockdown

**Figure 1:**
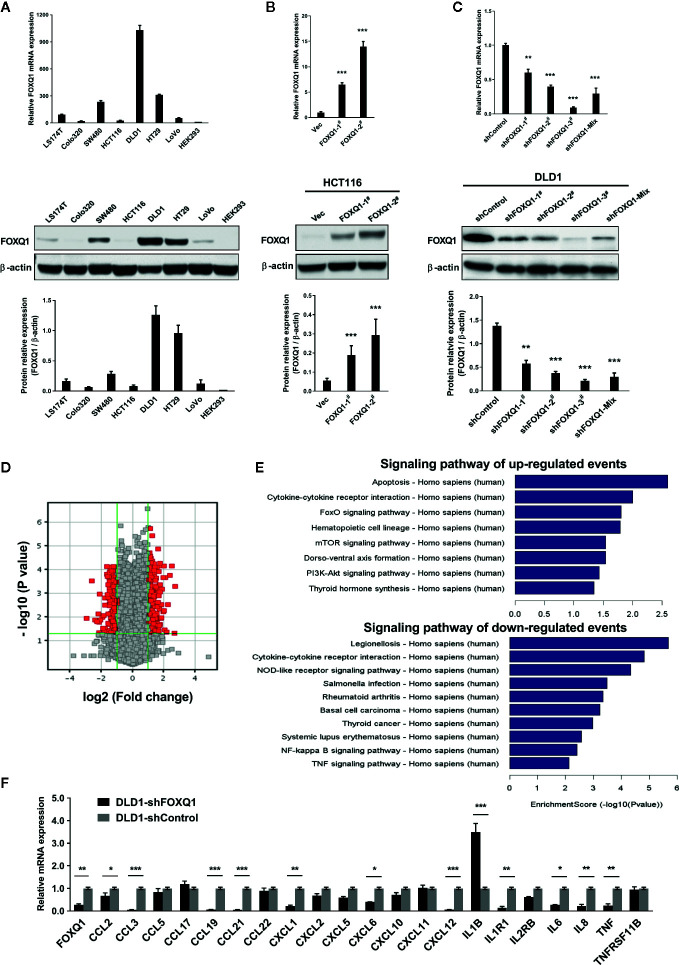
FOXQ1 modulates the expression of genes that affect the tumor microenvironment (TME) during colorectal cancer (CRC) tumorigenesis. **(A)** Relative endogenous FOXQ1 expression in 7 CRC cell lines; HEK293 was used as internal control. **(B)** Increased expression of FOXQ1 after transfection of HCT116 with either FOXQ1-1^#^ or FOXQ1-2^#^; HCT116 transfected with Vec was used as control. **(C)** Reduction in FOXQ1 after transfection of DLD1 with shFOXQ1-1^#^, shFOXQ1-2^#^, shFOXQ1-3^#^, or shFOXQ1-Mix; DLD1 transfected with shControl was used as control. **(D)** Volcano Plot visualizing differentially expressed genes (DEGs) between DLD1-shFOXQ1 and DLD1-shControl. The red point in the plot represents the DEGs with statistical significance. **(E)** Significant pathway analysis of up- and down-regulated DEG events; pathways are sorted in descending order based on enrichment score. Left, Pathway Name; right, bar graph representing the enrichment score [-log10 (*P* value)]. **(F)** Validation of the expression profiles of a subset of DEGs involved in cytokine-cytokine receptor interaction and the TNF signaling pathway by using qRT-PCR. ^*^ *P*<0.05, ^**^ *P*<0.01, ^***^ *P*<0.001 indicated a significant difference as compared to the control group (two-tailed, unpaired Student’s *t*-test). Bars represent mean ± S.E. of three independent experiments.

To elucidate functional roles for FOXQ1 in CRC, we generated CRC cell lines with stable FOXQ1 overexpression or shRNA. Among the CRC cell lines we tested, HCT116 had relatively low endogenous FOXQ1 expression, a feature already confirmed in several previous studies (10, 11, 14, 21, 25). Conversely, DLD1 had relatively high endogenous FOXQ1 expression. Both the HCT116 and DLD1 were CRC cell lines with malignant epithelial properties and originated from colorectal carcinoma. We therefore selected these two cell lines for over expression and knock down studies, respectively **(** **Figure 1A** **)**. RT-PCR and Western blotting assays verified the successful preparation of HCT116-FOXQ1-2^#^ **(**designated “HCT116-FOXQ1”; **Figure 1B** **)** and DLD1-shFOXQ1-3^#^ **(**designated “DLD1-shFOXQ1”; **Figure 1C** **)**. Transcriptional microarray analysis identified 431 DEGs (255 upregulated and 176 downregulated) between DLD1-shFOXQ1 and DLD1-shControl cells **(** **Figure 1D**, **Table S4**, GSE74223**)**. Furthermore, pathway analysis revealed several pathways related to oncogenesis. Notably, “Cytokine-cytokine receptor interactions” was identified as both significantly up- and downregulated, and the “TNF signaling pathway” was identified as downregulated, which suggests that FOXQ1 modulates the TME **(** **Figure 1E**, **Table S5**). To verify these effects of shFOXQ1, the expression profiles of a subset of 21 genes involved in TME modification were further validated by qRT-PCR. The results showed that mRNA expression of CCL2, CXCL12, IL6, IL8, and TNF, all of which are implicated in macrophage recruitment and inflammation, were dramatically downregulated in DLD1-shFOXQ1 compared with control cells **(** **Figure 1F**; CCL2 and IL6, *P*<0.05; IL8 and TNF, *P*<0.01; CXCL12, *P*<0.001**)**. The results also showed that mRNA expression of cytokines/chemokines relevant to chemotaxis of T cells, such as IL1B, was also dramatically upregulated in DLD1-shFOXQ1 compared with control cells **(** **Figure 1F**; IL1B, *P*<0.001**)**. These results suggest that FOXQ1 may play an important role in mediating TME modification during CRC tumorigenesis.

### Inhibition of FOXQ1 in CRC Cells Induces Suppressed Proliferation and Migration of CRC Cells *In Vitro*

**Figure 2:**
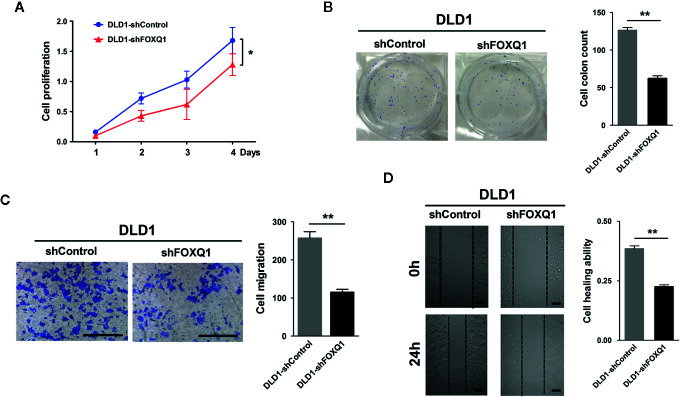
Inhibition of FOXQ1 induces suppressed proliferation and migration of colorectal cancer (CRC) cells *in vitro.* **(A)** FOXQ1 inhibition significantly supressed cell proliferation of DLD1-shFOXQ1 compared with control. **(B)** Significant decrease of clone formation in DLD1-shFOXQ1 compared with control. **(C)** Representative images of inhibition in migration DLD1-shFOXQ1 compared with control. **(D)** Representative images of inhibition in wound healing ability in DLD1-shFOXQ1 compared with control. All scale bars represent 100 μm. ^*^ *P*<0.05, ^**^ *P*<0.01 signify a significant difference between the indicated groups (two-tailed, unpaired Student’s *t*-test). *Bars* represent mean ± S.E. of three independent experiments.

To dissect the impact that FOXQ1 had on tumor progression and malignancy, we appraised its influence on cell proliferation and migration. We observed significant inhibition in both cell proliferation (**Figure 2A**; *P*<0.05) and clone formation (**Figure 2B**; *P*<0.01), and a concurrent decrease in migration (**Figure 2C**; *P*<0.01) and wound healing ability (**Figure 2D**; *P*<0.01), when FOXQ1 was suppressed in DLD1 cells. The *in vitro* results showed that inhibition of FOXQ1 could suppress the proliferation and migration of DLD1 cells.

### FOXQ1 Activates the Recruitment of HUVECs and Promotes Microvessel Morphogenesis *In Vitro*

**Figure 3:**
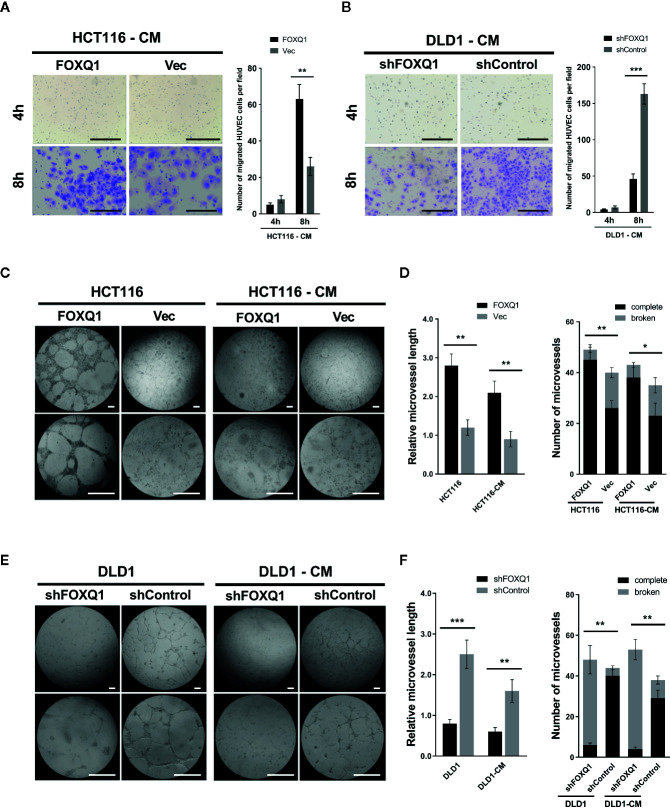
FOXQ1 promotes colorectal cancer (CRC) angiogenesis by activating recruitment of HUVECs and promotes microvessel morphogenesis *in vitro*. HUVEC migration was imaged at 4 h and 8 h after cell seeding into the Transwells. The number of HUVECs that had migrated was counted and normalized to that of the control group. **(A)** Treatment of HUVECs with CM from HCT116-FOXQ1 displayed a higher ability to recruit HUVECs than those with CM from HCT116-Vec. **(B)** Conversely, blocking expression of FOXQ1 in DLD1-shFOXQ1 resulted in less recruitment of HUVECs than that in the control group at 8 h. **(C)** Representative images of microvessel formation for HUVECs either co-cultured with either HCT116-FOXQ1 or HCT116-Vec, or CM of either HCT116-FOXQ1 or HCT116-Vec. **(D)** The relative microvessel length and number of complete and broken microvessels in HCT116-FOXQ1 cultures was compared with that in HCT116-Vec cultures. FOXQ1 overexpression significantly increased both the relative microvessel length and number of complete microvessels in CRC. **(E)** Representative images of microvessel formation for HUVECs co-cultured with either DLD1-shFOXQ1 or DLD1-shControl, or CM of either DLD1-shFOXQ1 or DLD1-shControl. **(F)** The relative microvessel length and number of complete and broken microvessels in DLD1-shFOXQ1 was compared with that in DLD1-shControl. FOXQ1 knockdown significantly decreased both the relative microvessel length and the number of complete microvessels in CRC. All scale bars represent 100 μm. ^*^ *P*<0.05, ^**^ *P*<0.01, ^***^ *P*<0.001 signify a significant difference between the indicated groups (two-tailed, unpaired Student’s *t*-test). CM, conditioned media.

Given the ability of FOXQ1 in CRC cells to modify the expression levels of genes related to the TME, we speculated that it might be involved in EC recruitment. Therefore, the effect of the FOXQ1 gene on the recruitment of HUVECs was detected using a Transwell system. HUVECs were cultured with CM collected from either HCT116-FOXQ1 or DLD1-shFOXQ1 cells. The results indicated that treatment of HUVECs with CM from HCT116-FOXQ1 for 8h displayed a higher ability to recruit HUVECs than those with CM from HCT116-Vec **(** **Figure 3A**; *P*<0.01**)**. Conversely, blocking expression of FOXQ1 in DLD1-shFOXQ1 for 8h resulted in less recruitment of HUVECs than that in the control group **(** **Figure 3B**; *P*<0.001**)**. Therefore, these results suggest that FOXQ1 mediates the recruitment of ECs, which comprises an initial step of angiogenesis.

Angiogenesis also entails the *de novo* formation of microvessels (3). To assess the function of FOXQ1 in regulating microvessel morphogenesis, *in vitro* microvessel formation assays were performed by co-culturing HUVECs with CRC cells or CM collected from CRC cells. The results show that HCT116-FOXQ1 cells elicited a strong angiogenic response and induced HUVECs to differentiate into microvessel structures; a similar angiogenic response was also observed with HCT116-FOXQ1-CM, though the effect was less obvious **(** **Figure 3C** **)**. Consistently, ectopic expression of FOXQ1 in HCT116 cells increased the microvessel length of HUVECs **(**left panel of **Figure 3D**; both *P*<0.01**)** and the abundance of intact microvessels **(**right panel of **Figure 3D**; *P*<0.01 for HCT116 cells and *P*<0.05 for CM**)**. Conversely, FOXQ1 knockdown in DLD1 resulted in a reduced angiogenic response and less HUVECs differentiating into microvessel structures either with DLD1-shFOXQ1 cells or DLD1-shFOXQ1-CM than in the control groups **(** **Figure 3E** **)**. Blocking the expression in DLD1 cells resulted in microvessels of reduced length **(**left panel of **Figure 3F**; *P*<0.001 and *P*<0.01 for DLD1 cells and CM, respectively**)** and decreased abundance **(**right panel of **Figure 3F**; both *P*<0.01**)**. These results suggest that FOXQ1 is essential for microvessel morphogenesis in CRC. The ability of the CM to confer a weaker angiogenic response than that of co-cultured cells implies that factors secreted by CRC cells can promote tumor angiogenesis, but that intercellular interactions between tumor cells and epithelial cells may also play important roles in promoting tumor angiogenesis.

### FOXQ1 Inhibition in CRC Cells Results in Inhibited Tumor Angiogenesis and Intratumoral Macrophage Infiltration *In Vivo*

**Figure 4:**
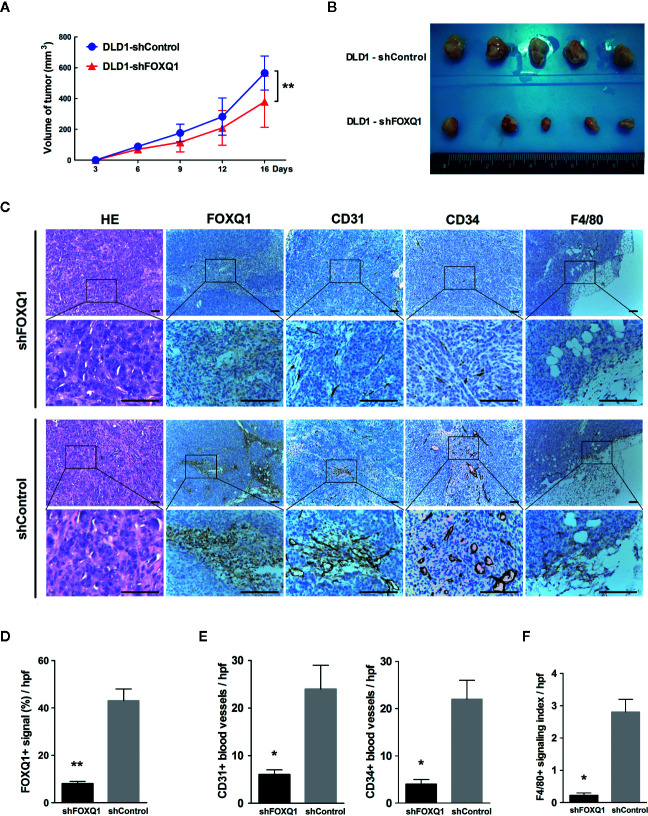
Inhibition of FOXQ1 results in inhibited tumor angiogenesis and intratumoral macrophage infiltration *in vivo.* Athymic nu-/nu-mice were implanted with 1 × 10^7^ DLD1-shFOXQ1 or DLD1-shControl cells subcutaneously (n = 5 per group); tumor volume was documented by caliper measurement. **(A)** FOXQ1 inhibition significantly suppressed the tumor proliferation capacity *in vivo*. **(B)** Resulting plugs were harvested and processed for IHC staining. **(C)** Representative HE staining images of tissue sections and IHC staining for FOXQ1; analysis of microvessel density (MVD) by IHC staining for CD31 or CD34; detection of the content of macrophages by IHC staining for F4/80. Scale bars represent 100 μm. **(D)** Reduction of FOXQ1 in dissected tumor tissue from the DLD1-shFOXQ1 group as determined by IHC staining quantification. **(E)** MVD was determined by counting the number of CD31^+^ or CD34^+^ vessels in tumor specimens; the number of CD31^+^ or CD34^+^ blood vessels was significantly decreased in DLD1-shFOXQ1 compared with DLD1-shControl. **(F)** Intratumoral macrophage quantification was determined by scoring for F4/80 staining intensity and distribution; DLD1 cells with downregulated FOXQ1 significantly decreased the number of infiltrating macrophages. 5 random hpf/section, 2-3 representative sections/plug, 5 plugs/group were scored for positive signal quantification. ^*^ *P*<0.05, ^**^ *P*<0.01 signifies a significant difference between the indicated groups (two-tailed, unpaired Student’s *t*-test). HE, Hematoxylin and eosin; IHC, immunohistochemical; hpf, high-powered fields.

To determine whether FOXQ1 affects tumor angiogenesis and intratumoral macrophage infiltration *in vivo*, tumor xenografts were obtained by implanting DLD1-shFOXQ1 or DLD1-shControl cells subcutaneously in nude mice. DLD1-shFOXQ1 resulted in approximately 2.68-fold decrease in tumor size relative to that of DLD1-shControl 16 days after implantation **(** **Figure 4A**; *P*<0.01). The sizes of dissected tumors reflected the differences in the tumor volumes **(** **Figure 4B** **)**. Furthermore, the morphological features of CRC were verified by H&E staining of xenograft tumor tissues **(** **Figure 4C** **)**, and immunostaining of FOXQ1 in dissected tumor tissues confirmed the reduction of FOXQ1 in DLD1-shFOXQ1 **(** **Figure 4D**; *P*<0.01**)**. Thus, these results suggest that inhibition of FOXQ1 suppresses the tumor proliferation capacity *in vivo*.

To determine the *in vivo* effect of FOXQ1 on tumor angiogenesis, MVD was evaluated by immunohistochemical staining of tumor specimens for the blood vessel markers CD31 and CD34, and the number of CD31^+^ **(**left panel of **Figure 4E**; *P*<0.05**)** and CD34^+^ **(**right panel of **Figure 4E**; *P*<0.05**)** blood vessels was significantly decreased in DLD1-shFOXQ1 compared with DLD1-shControl tumors. To determine the effect of FOXQ1 downregulation on macrophage chemotaxis, the macrophage content in transplanted CRC tissues was measured by staining for the mature murine macrophage marker F4/80. The results indicate that the TME of DLD1-shFOXQ1 tumors had significantly decreased numbers of infiltrating macrophages **(** **Figure 4F**; *P*<0.05**)**. Therefore, these results verify that FOXQ1 contributes to CRC angiogenesis and the CRC TME *in vivo*.

### FOXQ1 Inhibition in CRC Cells Downregulates Angiogenic Factors and the Chemoattractant CCL2 and Upregulates Angiogenic Inhibitors

**Figure 5:**
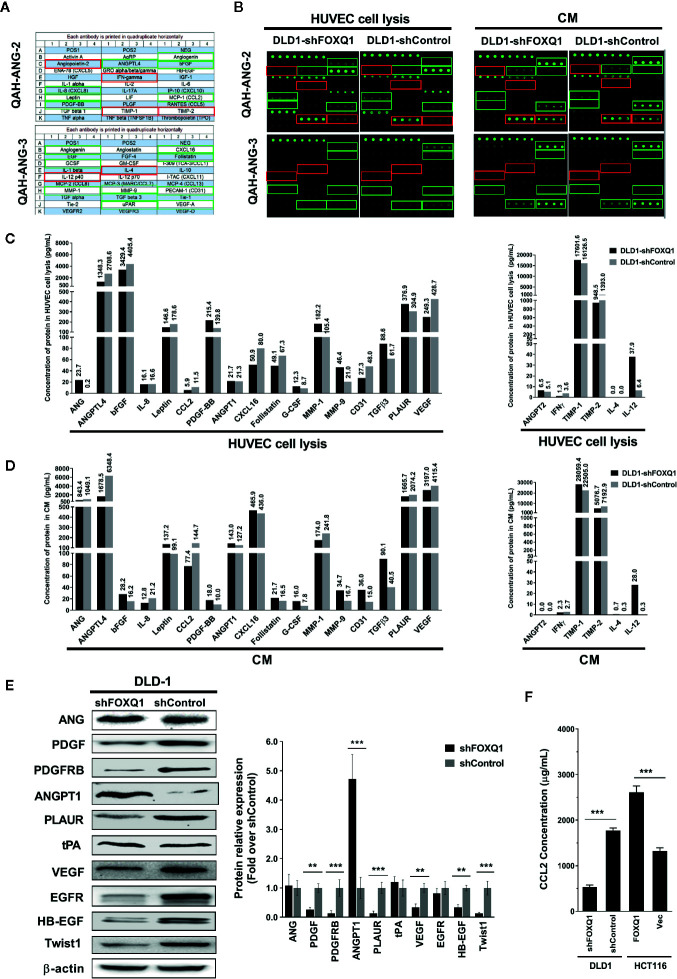
Inhibition of FOXQ1 induces downregulation of angiogenic factors and upregulation of angiogenic inhibitors. **(A)** Matrix distribution of 60 well-established angiogenic proteins on a Quantibody Human Angiogenesis Array (QAH-ANG-2 and QAH-ANG-3); each protein is distributed in quadruplicate horizontally. **(B)** Fluorescence detection of protein arrays for either cell lysate of HUVECs or conditioned medium (CM) of colorectal cancer (CRC) cells. CM was collected from either DLD1-shFOXQ1 or DLD1-shControl and added to HUVECs. After 48 h, HUVECs were harvested and lysed. Both the cell lysates and CM were collected, and the secretion of 60 angiogenic proteins were measured by hybridization with QAH-ANG-2 and QAH-ANG-3. The 17 green and 6 red rectangles on **(A, B)** show 17 angiogenesis factors and 6 angiogenic inhibitors within the detection range of the protein array. **(C)** Quantitative concentration of the 17 angiogenic factors and 6 angiogenic factors in cell lysates of HUVECs (pg/ml). **(D)** Quantitative concentration of the 17 angiogenic factors and 6 angiogenic factors in CM collected from either DLD1-shFOXQ1 or DLD1-shControl (pg/ml). The values above each bar on **(C, D)** represent quantitative concentration of corresponding protein, is the average of quadruplicates (n = 1 per group). **(E)** Western blot analyses for ANG, PDGF, PDGFRB, ANGPT1, PLAUR, tPA, VEGF, EGFR, HB-EGF, and Twist1 were performed with DLD1-shFOXQ1 and DLD1-shControl cell lysates. β-actin was used as the loading control. **(F)** Inhibition of FOXQ1 prevented the autocrine secretion of CCL2, whereas overexpression of FOXQ1 enhanced the secretion of CCL2, as determined by ELISA analysis. ^**^ *P*<0.01, ^***^ *P*<0.001 signifies a significant difference between the indicated groups (two-tailed, unpaired Student’s *t*-test). *Bars* represent mean ± S.E. of three independent experiments. HUVEC, human umbilical vein endothelial cell; CM, conditioned media.

As the secretion of regulatory factors and cytokines by tumor cells are known to promote angiogenesis and macrophage recruitment, we speculated that FOXQ1 might increase EC migration, microvessel morphogenesis, and macrophage recruitment by affecting the secretion of an array of regulatory factors and cytokines in CRC cells. Therefore, we quantified the expression and secretion of 60 well-established angiogenic factors in CM from CRC cells by protein array. Thirty-seven of the 60 angiogenic proteins were below the detectable concentration range, and the remaining 23 proteins (including 17 angiogenic factors and 6 angiogenic inhibitors) that were within the detection range were further analysed **(** **Figures 5A, B** **)**. Our results indicate that inhibition of FOXQ1 expression decreased the expression of important angiogenic factors in lysates from HUVECs that were cultured in CM from DLD1-shFOXQ1 (left panel of **(** **Figure 5C** **)**, as well as in CM from CRC cells **(**left panel of **Figure 5D** **)**, these decreased angiogenic factors include ANGPTL4, bFGF, Leptin, CCL2, CXCL16, Follistatin, and VEGF, most of which belong to EGF/PDGF pathway (34). In addition, CD31, a blood vessel marker, was decreased in HUVECs cultured with CM from DLD1-shFOXQ1 compared with those cultured with CM from DLD1-shControl **(**left panel of **Figure 5C** **)**. On the other hand, the inhibition of FOXQ1 expression promoted the secretion of 3 out of 6 angiogenic inhibitors including ANG-2, TIMP-1, and IL-12 both in HUVECs cell lysates **(**right panel of **Figure 5C** **)** and CM from CRC cells **(**right panel of **Figure 5D** **)**.

To further verify that FOXQ1 inhibition downregulates angiogenic factors while up-regulating angiogenic inhibitors, we performed Western blotting assays to evaluate the effect of FOXQ1 knockdown on the expression of selected proteins from the protein array (ANG, PDGF, PLAUR, ANGPT1, and VEGF), as well as additional proteins of the EGF/PDGF pathway that have been established to play important roles in tumor angiogenesis (PDGFRB, tPA, EGFR, and HB-EGF) (34). We also evaluated the expression of a downstream target gene previously shown to be regulated by FOXQ1 in CRC: Twist1 (11). Consistent with the findings from our protein array analysis and previous reports (10, 11), the protein levels of PDGF, PDGFRB, PLAUR, VEGF, EGFR, HB-EGF, and Twist1 were positively correlated with FOXQ1 expression, while the protein levels of ANG and tPA were not changed, and the protein level of ANGPT1 displayed the opposite trend **(** **Figure 5E** **)**. In summary, these results suggest that FOXQ1 inhibition in CRC cells induces downregulation of angiogenic factors while upregulating angiogenic inhibitors.

To further verify that FOXQ1 inhibition downregulates CCL2, a well-known macrophage chemoattractant (35), we performed ELISA analysis in CRC cells. The results confirm that shFOXQ1 prevented the autocrine secretion of CCL2 by DLD1 cells **(** **Figure 5F**; *P*<0.001**)**, whereas overexpression of FOXQ1 enhanced the secretion of CCL2 by HCT116 cells **(** **Figure 5F**; *P*<0.001**)**. These results indicate that FOXQ1 expression positively correlates with the ability of CRC cells to secrete CCL2, which could explain the increased macrophage infiltration in tumor cells **(** **Figure 4F** **)**.

### Twist1 Is Essential for FOXQ1-Mediated Macrophage Recruitment in CRC

**Figure 6:**
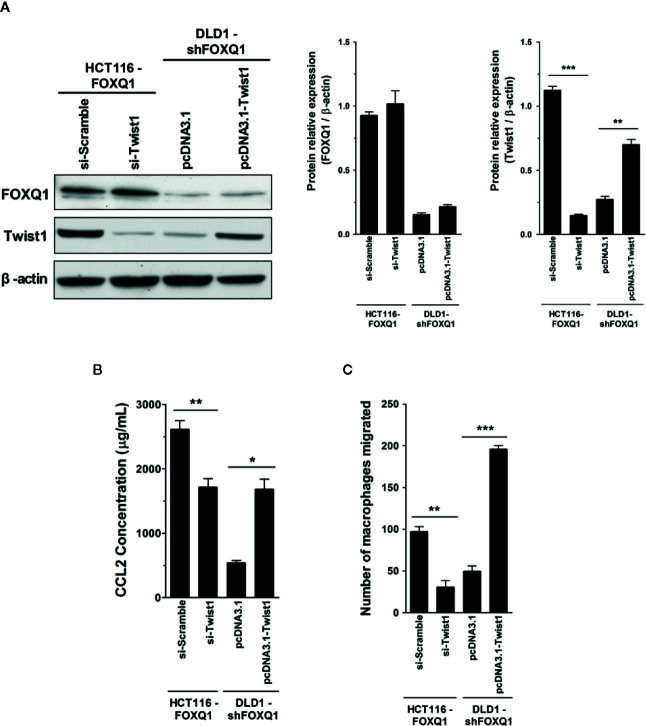
Twist1 is essential for FOXQ1-mediated macrophage recruitment in CRC. **(A)** HCT116-FOXQ1 cells were co-transfected either with si-Twist1 or si-Scramble; DLD1-shFOXQ1 was co-transfected either with pcDNA3.1-Twist1 or pcDNA3.1. After 48 h culture, Western blot analysis was performed for detection of FOXQ1 and Twist1 proteins. Twist1 knockdown abolished FOXQ1-mediated CCL2 upregulation, whereas upregulation of Twist1 rescued the decreased secretion of CCL2 induced by FOXQ1 knockdown. **(B)** Twist1 knockdown abolished FOXQ1-mediated CCL2 upregulation, whereas upregulation of Twist1 rescued the decreased secretion of CCL2 induced by FOXQ1 knockdown. ELISA analysis of CCL2 secretion was detected in CM. **(C)** Twist1 knockdown eliminated the FOXQ1-induced inhibition of macrophage recruitment, whereas upregulation of Twist1 rescued the decreased macrophage infiltration ability induced by FOXQ1. Chemotactic properties of CCL2 secreted by CRC cells on macrophage infiltration were determined by Transwell assay. ^*^ *P*<0.05, ^**^ *P*<0.01, ^***^ *P*<0.001 signifies a significant difference between the indicated groups (two-tailed, unpaired Student’s *t*-test). *Bars* represent mean ± S.E. of three independent experiments.

FOXQ1 is an established modulator of Twist1 expression and a regulator of cancer invasion and metastasis in CRC (11), and the role of Twist1-induced CCL2 in angiogenesis has been previously demonstrated (26). We therefore speculated that FOXQ1 might regulate macrophage infiltration by activating the Twist1/CCL2 axis. To further substantiate this hypothesis and to investigate the functional importance of the Twist1/CCL2 axis in macrophage infiltration, we measured CCL2 secretion in CM from HCT116-FOXQ1 and DLD1-shFOXQ1 cells that were co-transfected with si-Twist1 or pcDNA3.1-Twist1 **(** **Figure 6A** **)**. The results indicate that Twist1 knockdown abolishes FOXQ1-mediated CCL2 upregulation in HCT116-FOXQ1 cells **(** **Figure 6B**; *P*<0.01**)**. Conversely, upregulation of Twist1 increased CCL2 secretion in DLD1-shFOXQ1 cells **(** **Figure 6B**; *P*<0.05**)**. We next sought to explore the effect of Twist1 on FOXQ1-dependent macrophage infiltration induced by CM from CRCs. The results reveal that Twist1 knockdown eliminates the FOXQ1-induced inhibition of macrophage recruitment in HCT116-FOXQ1cells **(** **Figure 6C**; *P*<0.01**)**, whereas upregulation of Twist1 rescues the decreased macrophage infiltration ability induced by FOXQ1 **(** **Figure 6C**; *P*<0.001**)**. Taken together, these studies suggest that FOXQ1-mediated CCL2 secretion is dependent on Twist1 and that the Twist1/CCL2 axis is essential for FOXQ1-mediated macrophage recruitment in CRC.

### FOXQ1 Expression Is Positively Correlated With Twist1 and CCL2 Expression in Human CRC Tissues, and Their Positive Co-Expression Is Correlated With a Lower 8-Year Survival Rate

**Figure 7:**
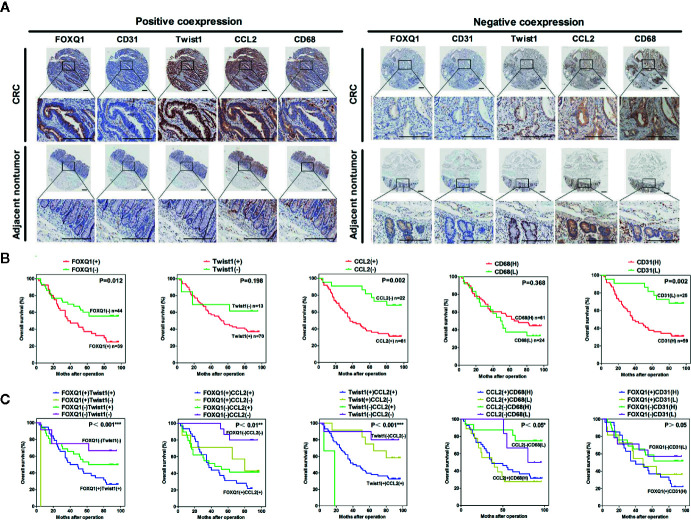
FOXQ1 is associated with enhanced cancer angiogenesis and macrophage recruitment in human colorectal cancer (CRC). **(A)** Representative immunohistochemical images of FOXQ1, CD31, Twist1, CCL2, and CD68 positive co-expression and negative co-expression in human CRC tissues and adjacent nontumorous tissues. Scale bars represent 200 μm. **(B)** Kaplan-Meier survival analysis of CRC patients with positive expression of either FOXQ1 or CCL2 had shorter overall survival than those with negative expression of FOXQ1 or CCL2 in a cohort of 83 CRC patients. **(C)** Kaplan-Meier survival analysis of CRC patients with positive co-expression of both FOXQ1/Twist1, or FOXQ1/CCL2, Twist1/CCL2, or CCL2/CD68 had the shortest overall survival times compared with the corresponding single negative or double negative groups in the cohort of 83 CRC patients. ^*^ *P*<0.05, ^**^ *P*<0.01, ^***^ *P*<0.001 signifies a significant difference between the indicated groups (log-rank test).

To verify the clinical relevance of our findings, we evaluated the expression of FOXQ1, CD31, Twist1, CCL2, and CD68 in human CRC tissue biopsies (cohort, n=83). IHC results showed that FOXQ1, Twist1, and CCL2 each were significantly upregulated in CRC tissues compared with adjacent nontumorous tissues, and that CD31 and CD68 were moderately upregulated **(** **Figure 7A** **)**. Furthermore, the overexpression of FOXQ1 was significantly correlated with lymph node metastasis or higher TNM stage **(** **Table 1** **)**, which is consistent with our previous study (12). Further analysis verified the statistically significant correlation between FOXQ1 and Twist1 (*P*<0.01) **(** **Table 2** **)**. Twist1 expression was also positively correlated with CCL2 expression in cohort CRC tissues **(** **Table 3** **)**, but not CD68 expression **(** **Table 4** **)**. Finally, Kaplan-Meier survival analysis was performed to further validate the role of FOXQ1 in promoting tumor angiogenesis and TME modification. The results show that CRC patients with positive expression of either FOXQ1 (*P* = 0.012) or CCL2 (*P* = 0.002) had shorter overall survival than those with negative expression of FOXQ1 or CCL2 **(** **Figure 7B** **)**. Similarly, CRC patients with higher expression levels of CD31 had shorter overall survival than those with lower expression levels of CD31 (*P* = 0.002). Furthermore, CRC patients with positive co-expression of both FOXQ1/Twist1 (*P*<0.001), or FOXQ1/CCL2 (*P*<0.01), Twist1/CCL2 (*P*<0.001), or CCL2/CD68 (*P*<0.05) had the shortest overall survival times compared with the corresponding single negative or double negative groups **(** **Figure 7C** **)**. Thus, expression of FOXQ1 and its co-regulated proteins may have prognostic relevance in CRC.

**Table 1:** Correlation between FOXQ1 expression and clinicopathological characteristics of colorectal cancers (CRCs) in cohort of human CRC tissues.

Clinicopathological variables		Cohort tumor FOXQ1 expression		*P* Value
		Negative (n=44)	Positive (n=39)	
Age		69.28 (12.36)	70.54 (9.41)	
Gender	Female	19	21	> 0.05
	Male	25	18	
Maximal tumor size	≤10	10	9	>0.05
	>10	34	30	
Lymphatic metastasis	Absent	32	20	<0.05*
	Present	12	19	
Tumor differentiation	I–II	21	24	>0.05
	III–IV	23	15	
TNM stage	I–II	17	6	<0.05*
	III-IV	27	33	
AJCC clinical stage according to 7th issue	1 & 2A	22	24	>0.05
	3 & 3B	12	15	
Histological subtype	Adenocarcinoma (information about histological subtypes unavailable)	16	18	>0.05
	Tubular adenocarcinoma	23	19	
	Tubular adenocarcinoma with partial mucinous adenocarcinoma,	4	0	
	Mucinous adenocarcinoma	0	1	
	Papillary adenocarcinoma	0	1	
	Adenocarcinoma with squamous epithelial metaplasia	1	0	

AJCC, American Journal of Critical Care. *P<0.05.

**Table 2:** Correlation analysis of FOXQ1 and Twist1, CCL2, or CD31 expression in cohort (n=83) colorectal cancer (CRC) tissues.

		FOXQ1		*x* ^2^	*P* value
		negative (n=44)	positive (n=39)		
Twist1	negative (n=13)	12	1	9.56	<0.01^**^
	positive (n=70)	32	38		
CCL2	negative (n=22)	15	7	2.77	>0.05
	positive (n=61)	29	32		
CD31	low (n=24)	16	8	2.53	>0.05
	high (n=59)	28	31		

^**^P<0.01.

**Table 3:** Correlation analysis of Twist1 and CCL2 expression in cohort (n=83) colorectal cancer (CRC) tissues.

		Twist1		*x* ^2^	*P* value
		negative (n=13)	positive (n=70)		
CCL2	negative (n=22)	10	12	17.16	<0.01^**^
	positive (n=61)	3	58		

^**^P<0.01.

**Table 4:** Correlation analysis of CCL2 and CD68 expression in cohort (n=83) colorectal cancer (CRC) tissues.

		CCL2		*x* ^2^	*P* value
		negative (n=22)	positive (n=61)		
CD68	low (n=22)	3	20	2.96	>0.05
	high (n=61)	19	41		

## Discussion

Tumor angiogenesis is an important component of cancer development, involving a multistep process of EC migration and tubular formation (36). Macrophages are one of the main infiltrating cell groups in the cancer stroma, promoting the progression of tumors by releasing growth and angiogenic factors (37, 38). Angiogenesis and macrophage recruitment are closely related to cancer progression, and these two biological processes share common pathways (39).

In this study, we found that FOXQ1 inhibition in CRC cells results in suppressed proliferation and migration of CRC cells *in vitro* **(** **Figure 2** **)**, this result was consistent with ours (12) and other previous reports in CRC (11, 21). Furthermore, the impact of FoxQ1 on promoting tumor cell proliferation was also well established in other solid tumors including ovarian cancer (16), neuroblastoma (20), lung cancer (22), gastric cancer (23), and liver cancer (40). We also demonstrated that CRC cells with decreased FOXQ1 expression, as well as CM from these cells, can activate migration and microvessel morphogenesis of HUVECs *in vitro* **(** **Figure 3** **)**. The effect of FOXQ1 knockdown was further confirmed in *in vivo* experiments, demonstrating that FOXQ1 inhibition in CRC cells results in slower xenograft tumor growth and angiogenesis. Interestingly, FOXQ1 inhibition in CRC cells also evidently reduced recruitment of macrophages in our mouse model **(** **Figure 4** **)**. A limitation, however, should be taken into account when the findings of the present study are interpreted. *in vivo* study was only performed by using FOXQ1 knockdown DLD1 cells, an independent xenograft study by using FOXQ1 overexpressed CRC cell lines would make the results more convincing.

To evaluate which angiogenesis and macrophage recruitment factors are induced by FOXQ1 in CRC cells, we performed protein array analysis. The inhibition of FOXQ1 expression in CRC cells caused a pronounced decrease in the secretion of several angiogenic factors, whereas it caused a significant increase in the endogenous angiogenic inhibitor ANGPT1. Of particular interest, our results indicated VEGF was downregulated both in CM from DLD1-shFOXQ1 cells and in HUVECs cultured with this CM **(** **Figures 5C–E** **)**, this result was consistent with previous report, in which identified VEGFA as a candidate target gene of FOXQ1 (10). VEGF is the most crucial factor involved in angiogenesis, controlling the early steps that trigger the angiogenic cascade, which promotes EC migration and proliferation (41), VEGF play its roles *via *VEGF receptor 2 (VEGFR2), VEGF/VEGFR2 signalling is a key signalling event in angiogenesis and vascular permeability (42), further studies should be performed to demonstrate the effect of FOXQ1 in VEGF/VEGFR2 signalling.

Several additional critical angiogenic factors were also decreased with FOXQ1 inhibition in CRC cells, including PDGF and its receptor PDGFRB, as well as HB-EGF, one of the critical ligands of EGFR (6). Most of these angiogenic factors are molecular components of the EGF/PDGF pathway, which plays an important role in activating tumor angiogenesis (5). These results suggest that FOXQ1 overexpression in CRC cells can promote tumor angiogenesis either by promoting secretion of tumor cell-produced angiogenic factors mainly in the EGF/PDGF pathway, while reducing the expression of angiogenic inhibitors **(** **Figure 5** **)**. This notion was supported by our data showing that expression of the vascular marker CD31 were also decreased in EC cultured in CM from FOXQ1-inhibited CRC cells **(** **Figure 5C** **)**.

It has been well established that the TME plays an important role in tumorigenesis (4, 43). Macrophages, which are the most abundant immune-related stromal cells in the TME (37, 43), are key orchestrators of the TME, directly affecting neoplastic cell growth, angiogenesis, and extracellular matrix remodeling (44). The pro-tumor role of macrophages in CRC is controversial. Some studies have indicated that macrophages in CRC appear to have antitumor activity and are associated with improved disease-free survival (45). In contrast, other studies demonstrate that macrophages in CRC often display an alternatively activated phenotype, promoting tumor progression and disease aggressiveness (46, 47), and are associated with poor prognosis in CRC patients (48). In addition, cancer cells can actively modulate macrophages in the TME to enhance cancer development and metastasis (49). In the current study, we found that FOXQ1 inhibition in CRC cells results in inhibited intratumoral macrophage infiltration *in vivo*. This observation revealed that FOXQ1 accelerates tumor growth not only by strengthening tumor angiogenesis but also by promoting macrophage recruitment **(** **Figure 4** **)**.

Given the effect of FOXQ1 on macrophage recruitment, we sought to evaluate a potential role for CCL2, a well-established macrophage chemoattractant, in FOXQ1-dependent CRC secretion (26). CCL2 was decreased both in CM from DLD1 cells with FOXQ1 inhibition and HUVECs cultured with this CM **(** **Figures 5C, E** **)**, and CCL2 secretion positively correlated with FOXQ1 expression in CRC cells **(** **Figure 5F** **)**. We also evaluated the effects of FOXQ1 expression on Twist1, which has been established as a direct target of FOXQ1 in cancers including CRC (11, 50, 51). Evidence suggests that Twist1 can directly activate CCL2 and promote angiogenesis by increasing macrophage recruitment (26). Twist1 overexpression can also increase the synthesis of VEGF, promote vascular expansion and permeability, and accelerate tumor progression (52). Our macrophage migration results demonstrated that FOXQ1 overexpression in CRC cells can stimulate the production of CCL2, thus promoting macrophage infiltration within the TME, whereas Twist1 knockdown reversed the increased CCL2 expression and macrophage infiltration induced by FOXQ1 overexpression. In contrast, knockdown of FOXQ1 in CRC cells decreased CCL2 expression and macrophage infiltration, whereas upregulation of Twist1 rescued the decreased macrophage infiltration and CCL2 expression induced by FOXQ1 knockdown **(** **Figure 6** **)**. Taken together, our data suggest that FOXQ1-mediated macrophage infiltration is dependent on the Twist1/CCL2 axis.

To further confirm that FOXQ1 promotes tumor angiogenesis and modifies the TME in CRC, tumor tissues from 83 patients diagnosed with CRC were used to evaluate the expression of FOXQ1, Twist1, CCL2, CD68, and CD31. IHC results showed that the expression of FOXQ1, Twist1, and CCL2 in CRC tissues was significantly higher than that in para-carcinoma tissues **(** **Figure 7A** **)**. The expression of FOXQ1 was demonstrated to be positively correlated with lymph node metastasis and TNM stage **(** **Table 1** **)**. Although we did not found any differential expression pattern of FOXQ1 in CRC subtypes statistically in present work. A cohort study based on larger sample needed to be conduct to characterize the potential relationship between expression pattern of FOXQ1 and CRC histologic subtypes **(** **Table 1** **)**. Furthermore, there was a positive correlation between FOXQ1 and the expression of Twist1 **(** **Table 2** **)**; and between Twist1 and the expression of CCL2 **(** **Table 3** **)**. These studies suggest that overexpression of the FOXQ1-induced Twist1/CCL2 axis plays an important role in promoting CRC macrophage infiltration. The role of Twist1-induced CCL2 in angiogenesis has been elucidated previously (26). Furthermore, CCL2 was originally identified as a tumor-derived chemotactic factor for macrophages (53), and the levels of tumor-derived CCL2 significantly correlate with macrophage density and the depth of invasion in various cancers (54). Moreover, experimental studies using xenotransplanted tumors have revealed the involvement of the CCL2/CCR2 axis in cancer metastasis (55). In present study, Kaplan-Meier survival analysis further confirmed that FOXQ1-induced Twist1/CCL2 axis is closely associated with a lower 8-year survival in CRC patients **(** **Figures 7B, C** **)**. However, we failed to find a positive correlation between FOXQ1 and the endothelial marker CD31 **(** **Table 2** **)**, and their co-expression was not associated with poorer prognosis in CRC patients **(** **Figure 7C** **)**. Our interpretation of these findings is that CD31 is a universal marker of EC but not a specific marker of tumor vessels. More specific markers for tumor vessels will be beneficial in confirming these findings.

**Figure 8:**
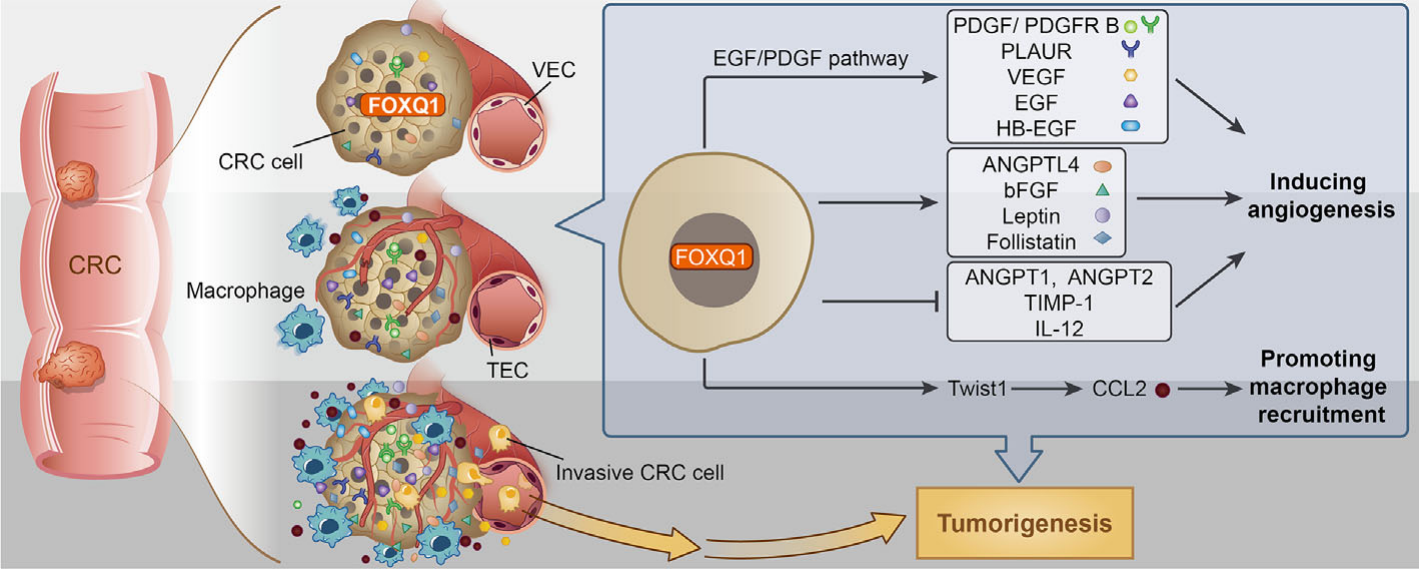
Potential mechanism for Forkhead box Q1 (FOXQ1) induced angiogenesis and macrophage infiltration in colorectal cancer (CRC) tumorigenesis. We speculate that FOXQ1 can promote the migration of endothelial cell in tumor microenvironment (TME), improve microvessel morphogenesis, as well as strengthen intercellular interactions between tumor and epithelial cells mainly through activation of EGF/PDGF pathway. We also propose that FOXQ1 can promote macrophage infiltration in TME by activating the Twist1/CCL2 axis. Thus, the combined mechanisms of FOXQ1 described in this study support a dual role of FOXQ1 in promoting CRC tumorigenesis.

In summary, there are two major steps during the pathological angiogenic process. First, tumor vascular formation involves the migration and differentiation of endothelial progenitors through vasculogenesis (56). Second, host inflammatory cells, including macrophages, infiltrate tumor tissues, alter the microenvironment, and promote tumor angiogenesis (57). Here, we confirmed that FOXQ1 directly contributes to both major steps. The essential role of FOXQ1-induced angiogenesis and macrophage recruitment in CRC is likely to be related to its ability to promote the migration of ECs and macrophages in the TME through activation of the EGF/PDGF pathway and the Twist1/CCL2 axis, respectively **(** **Figure 8** **)**, thus supporting a dual role for FOXQ1 in promoting CRC progression. Based on our findings, FOXQ1 may serve as a therapeutic target for CRC treatment by inhibiting tumor angiogenesis and reducing macrophage recruitment.

## Data Availability Statement

Publicly available datasets were analyzed in this study, these can be found in the NCBI Gene Expression Omnibus (GSE74223).

## Ethics Statement

The animal study was reviewed and approved by Institutional Animal Care and Use Committee, the First People’s Hospital of Yunnan Province (Yunnan, China).

## Author Contributions

HT and QG designed the project, analyzed data, and wrote the manuscript. JZ performed xenograft model assay, IHC and MVD analysis. XB performed microvessel formation assay. K-LY generated stable FOXQ1 overexpress/knockdown cell lines. J-HL and D-YL performed *in vitro* macrophages migration experiments. L-PW and J-LW carried out HUVECs migration assay, Elisa, and Western blotting. HT analyzed colorectal tissue microarray data. All authors contributed to the article and approved the submitted version.

## Funding

This study was founded by the National Natural Science Foundation of China [Grants 81502556, 81460463 and 81260323]; the Medical Academic Talents Cultivation Foundation for Health Commission of Yunnan Province [Grant D-201642]; the Foundation of Kunming Key Laboratory of Tumor Molecular and Immune Prevention [2018-1-A-17334]; and the Yunnan Digestive Endoscopy Clinical Medical Center Foundation for Health Commission of Yunnan Province [2X2019-01-02].

## Conflict of Interest

The authors declare that the research was conducted in the absence of any commercial or financial relationships that could be construed as a potential conflict of interest.
