# Supplementary material for: Forkhead Box Q1 Is Critical to Angiogenesis and Macrophage Recruitment of Colorectal Cancer
Source: Front Oncol. 2020 Nov 30;10:564298. doi: 10.3389/fonc.2020.564298 (PMC7734287; doi:10.3389/fonc.2020.564298)
Supplement: Supplementary file 1 [file Table_1.docx]

Supplementary Tables

**TABLE S1.** Sequences of siRNAs targeting the human FOXQ1 sequence.

|  | siRNA Sense ( 5’ –3’) | Antisense (5’ –3’) |
| --- | --- | --- |
| FOXQ1-siRNA-1# | CUCCAUCAAACGUGCCUUAdTdT | UAAGGCACGUUUGAUGGAG dTdT |
| FOXQ1-siRNA-2# | GCUAUUGACCGAUGCUUCA dTdT | UGAAGCAUCGGUCAAUAGC dTdT |
| FOXQ1-siRNA-3# | CUUGAAAGCAAGUGUGAUU dTdT | AAUCACACUUGCUUUCAAGdTdT |
| Scramble-siRNA | UUCUCCGAACGUGUCACGU dTdT | ACGUGACACGUUCGGAGAA dTdT |

**TABLE S2.** Nucleotide sequences of the primers used for plasmid construction.

| ID | AgeI | Sense | Loop | Antisense | Terminator | EcoRI |
| --- | --- | --- | --- | --- | --- | --- |
| shRNA-1#-S | 5’-CCGG | CTCCATCAAACGTGCCTTA | CTCGAG | TAAGGCACGTTTGATGGAG | TTTTT | G-3’ |
| shRNA-1#-A | 3’- | GAGGTAGTTTGCACGGAAT | GAGCTC | ATTCCGTGCAAACTACCTC | AAAAA | CTTAA-5’ |
| shRNA-2#-S | 5’-CCGG | GCTATTGACCGATGCTTCA | CTCGAG | TGAAGCATCGGTCAATAGC | TTTTT | G-3’ |
| shRNA-2#-A | 3’- | CGATAACTGGCTACGAAGT | GAGCTC | ACTTCGTAGCCAGTTATCG | AAAAA | CTTAA-5’ |
| shRNA-3#-S | 5’-CCGGT | CTTGAAAGCAAGTGTGATT | CTCGAG | AATCACACTTGCTTTCAAG | TTTTT | G-3’ |
| shRNA-3#-A | 3’-AA | GAACTTTCGTTCACACTAA | GAGCTC | TTAGTGTGAACGAAAGTTC | AAAAA | CTTAA-5’ |
| shRNA-Cr-S | 5’-CCGG | TTCTCCGAACGTGTCACGT | CTCGAG | ACGTGACACGTTCGGAGAA | TTTTT | G-3’ |
| shRNA-Cr-A | 3’- | AAGAGGCTTGCACAGTGCA | GAGCTC | TGCACTGTGCAAGCCTCTT | AAAAA | CTTAA-5’ |

**TABLE S3.** Oligonucleotide sequences of primers used for qRT-PCR analysis.

| Gene | Sequence of Primer (5’- 3’) | |
| --- | --- | --- |
|  | Forward Primer | Reverse Primer |
| FOXQ1 | GGCAACGGGCTACAGCTTTA | GGCACCCCACATACATAATCAA |
| GAPDH | TGACTTCAACAGCGACACCCA | CACCCTGTTGCTGTAGCCAAA |
| CCL2 | CAGCCAGATGCAATCAATGCC | TGGAATCCTGAACCCACTTCT |
| CCL3 | CTGCTCAGAATCATGCAGGTC | ACTGGCTGCTCGTCTCAAAG |
| CCL5 | TCCCACAGGTACCATGAAGGTC | GCAATGTAGGCAAAGCAGCAG |
| CCL17 | TGAGGACTGCTCCAGGGATG | AACGGTGGAGGTCCCAGGTA |
| CCL19 | AGCCTGCTGGTTCTCTGGACT | TGCAGCCATCCTTGATGAGAA |
| CCL21 | GCCACACTCTTTCTCCTGCTTT | ACTCTCCCTCCTCGGTCTCTCT |
| CCL22 | GGTATTTGAACCTGTGGAATTGGAG | CAGGCCCTGGATGACACTGA |
| CXCL1 | CAAACCGAAGTCATAGCCACAC | AGTGAGCTTCCTCCTCCCTTCT |
| CXCL2 | CCACACTCAAGAATGGGCAGA | CCTTCAGGAACAGCCACCAA |
| CXCL5 | GAGAGCTGCGTTGCGTTTG | TTTCCTTGTTTCCACCGTCCA |
| CXCL6 | CGCTGGTCCTGTCTCTGC | GTTTTTCTTGTTTCCACTGTCC |
| CXCL10 | GACATATTCTGAGCCTACAGCAGAG | GTTGATTACTAATGCTGATGCAGGT |
| CXCL11 | GCTGTGATATTGTGTGCTACAGTTG | TTGGGTACATTATGGAGGCTTTC |
| CXCL12 | CCCTGCTTACCCGCAAAA | CTTCAGAGGCAATCACAAAACC |
| IL1B | TGAAGCAGCCATGGCAGAAG | GGTCGGAGATTCGTAGCTGGA |
| IL1R1 | TTTGACACACAGACTACAGCCAGA | GTTTCCAAATTGGCTTGCTTTC |
| IL2RB | CTCAGAGTGCTTGGCTGCTGTA | GCCTCGGACTCCAGGGTTTA |
| IL6 | AGTTTCCTGCAATGGATCAAGG | CTGCTTATCTGAAGGTGTTGCAC |
| IL8 | ATGACTTCCAAGCTGGCCGTGGCT | TCTCAGCCCTCTTCAAAAACTTCTC |
| TNF | TGCTTGTTCCTCAGCCTCTT | CAGAGGGCTGATTAGAGAGAGGT |
| TNFRSF11B | ATTTGCCTGGCACCAAAGT | GAAGGTGAGGTTAGCATGTCCA |

**TABLE S4.** List of top 20 up- and downregulated genes identified between DLD1-shFOXQ1 and DLD1-shControl cells in descending order with a fold change ≥2.0. or ≤-2.0.

|  | **Gene Symbol** | **Fold Change** | **mRNA Accession** | **Description** |
| --- | --- | --- | --- | --- |
| up | GPA33 | 6.626402 | NM_005814 | glycoprotein A33 |
|  | DNMBP-AS1 | 6.349088 | NR_024130 | DNMBP antisense RNA 1, non-coding RNA |
|  | ANGPTL4 | 5.696401 | NM_139314 | angiopoietin-like 4 |
|  | DUOX2 | 5.223868 | NM_014080 | dual oxidase 2 |
|  | IL1B | 4.755472 | NM_000576 | interleukin 1, beta |
|  | BLNK | 4.511446 | NM_013314 | B-cell linker |
|  | VTCN1 | 4.171324 | NM_024626 | V-set domain containing T cell activation inhibitor 1 |
|  | SFTPB | 4.070699 | NM_000542 | surfactant protein B |
|  | SHE | 4.028845 | NM_001010846 | Src homology 2 domain containing E |
|  | _ | 3.957717 | DB335107 | SYNOV4 Homo sapiens cDNA clone SYNOV4009599 3', mRNA sequence |
|  | LOC100131581 | 3.913005 | AK092544 | cDNA FLJ35225 fis, clone PROST2001116 |
|  | REPS2 | 3.894184 | NM_004726 | RALBP1 associated Eps domain containing 2 |
|  | C7orf60 | 3.657602 | NM_152556 | chromosome 7 open reading frame 60 |
|  | DUOXA2 | 3.601023 | NM_207581 | dual oxidase maturation factor 2 |
|  | C5orf4 | 3.594347 | NM_032385 | chromosome 5 open reading frame 4 |
|  | LOC283050 | 3.530351 | NR_024431 | uncharacterized LOC283050, transcript variant 1, non-coding RNA |
|  | LOC100132354 | 3.422083 | NR_024478 | uncharacterized LOC100132354, non-coding RNA |
|  | ITM2B | 3.385245 | NM_021999 | integral membrane protein 2B |
|  | ALDOC | 3.37901 | NM_005165 | aldolase C, fructose-bisphosphate |
|  | C15orf48 | 3.370889 | NM_032413 | chromosome 15 open reading frame 48 |
| down | TCF24 | **-**7.699125 | NM_001193502 | transcription factor 24 |
|  | MORN5 | **-**6.097624 | NM_198469 | MORN repeat containing 5 |
|  | GRK1 | **-**5.3753 | NM_002929 | G protein-coupled receptor kinase 1 |
|  | SPOCD1 | **-**4.863297 | NM_144569 | SPOC domain containing 1 |
|  | GDF6 | **-**4.438107 | NM_001001557 | growth differentiation factor 6 |
|  | SLC39A8 | **-**4.372665 | NM_001135147 | solute carrier family 39 (zinc transporter), member 8 |
|  | FAM48B2 | **-**4.206 | NM_001136233 | family with sequence similarity 48, member B2 |
|  | SERPINI1 | **-**3.898295 | NM_005025 | serpin peptidase inhibitor, clade I (neuroserpin), member 1 |
|  | MYBPH | **-**3.67658 | NM_004997 | myosin binding protein H |
|  | VPS53 | **-**3.553322 | NM_001128159 | vacuolar protein sorting 53 homolog |
|  | C3 | **-**3.442721 | NM_000064 | complement component 3 |
|  | XIAP | **-**3.427454 | U45880 | X-linked inhibitor of apotosis protein |
|  | ZNF879 | **-**3.426777 | NM_001136116 | zinc finger protein 879 |
|  | CCL20 | **-**3.145884 | NM_004591 | chemokine (C-C motif) ligand 20 |
|  | FMR1NB | **-**3.13995 | NM_152578 | fragile X mental retardation 1 neighbor |
|  | GNN | **-**3.113418 | NR_027249 | Grp94 neighboring nucleotidase pseudogene, non-coding RNA |
|  | LOC400568 | **-**3.076233 | BC043554 | cDNA clone IMAGE:5176687 |
|  | DGCR14 | **-**3.033284 | NM_022719 | DiGeorge syndrome critical region gene 14 |
|  | S100A8 | **-**2.988219 | NM_002964 | S100 calcium binding protein A8 |
|  | RN28S1 | **-**2.977916 | NR_003287 | RNA, 28S ribosomal 1, ribosomal RNA |
|  | FOXQ1 | -2.340340 | NM_033260.3 | Forkhead box Q1 |

**TABLE S5. Subset of genes involved in FOXQ1-targeted pathways identified by pathway analysis.**

| **Pathway** | **Genes** | **Enrichment Score** | **FDR** |
| --- | --- | --- | --- |
| **Up-regulated events** |  |  |  |
| Apoptosis | CASP10//IL1B//IL1R1//IL3RA//PRKACB | 2.5925 | 0.7182 |
| Cytokine-cytokine receptor interaction | ACVR2B//EGFR//IL1B//IL1R1//IL2RB//IL3RA//IL4//PDGFRB | 2.0017 | 1.0000 |
| FoxO signaling pathway | CCNG2//EGFR//FBXO32//S1PR4//SGK2 | 1.8017 | 1.0000 |
| Hematopoietic cell lineage | IL1B//IL1R1//IL3RA//IL4 | 1.7900 | 1.0000 |
| mTOR signaling pathway | DDIT4//RICTOR//TSC1 | 1.5428 | 1.0000 |
| Dorso-ventral axis formation | EGFR//PIWIL3 | 1.5423 | 1.0000 |
| PI3K-Akt signaling pathway | DDIT4//EGFR//IL2RB//IL3RA//IL4//PDGFRB//SGK2//TSC1 | 1.4303 | 1.0000 |
| Thyroid hormone synthesis | DUOX2//DUOXA2//PRKACB | 1.3422 | 1.0000 |
| **Down-regulated events** |  |  |  |
| Legionellosis | C3//CXCL1//CXCL2//HSPA1B//IL8//TNF | 5.6814 | 0.0006 |
| Cytokine-cytokine receptor interaction | CCL20//CXCL1//CXCL2//GDF6//IL17RA//IL8//LTB//TNF//TNFRSF11B//TPO | 4.8088 | 0.0022 |
| NOD-like receptor signaling pathway | CARD9//CXCL1//CXCL2//IL8//TNF | 4.3323 | 0.0044 |
| Salmonella infection | ARPC4//CXCL1//CXCL2//IL8//MYH9 | 3.4792 | 0.0233 |
| Rheumatoid arthritis | CCL20//CXCL1//IL8//LTB//TNF | 3.3432 | 0.02550 |
| Basal cell carcinoma | CTNNB1//GLI1//TP53//WNT16 | 3.2310 | 0.0275 |
| Thyroid cancer | CTNNB1//TP53//TPM3 | 2.9659 | 0.0434 |
| Systemic lupus erythematosus | C3//HIST1H2BG//HIST1H3H//HIST1H4D//TNF | 2.5662 | 0.0954 |
| NF-kappa B signaling pathway | CXCL2//IL8//LTB//TNF | 2.4180 | 0.1192 |
| TNF signaling pathway | CCL20//CXCL1//CXCL2//TNF | 2.1262 | 0.2101 |

FDR: False discovery rate.
